# Exploring the association between mental imagery, sensory sensitivity, and autistic traits in autistic and non-autistic adults

**DOI:** 10.1038/s41598-026-38574-9

**Published:** 2026-02-25

**Authors:** Rebecca Taylor, Petroc Sumner, Krish D. Singh, Catherine R. G. Jones

**Affiliations:** 1 Wales Autism Research Centre, School of Psychology, Cardiff University, Cardiff, UK; 2 School of Psychology, Cardiff University Brain Research Imaging Centre (CUBRIC), Cardiff University, Cardiff, UK

**Keywords:** Neuroscience, Psychology, Psychology

## Abstract

Mental imagery vividness varies between individuals. Low levels of mental imagery have been associated with high levels of autistic traits, whilst autistic traits are known to positively correlate with sensory sensitivities. This would predict a negative correlation between sensory sensitivity and imagery. However, one recent study has suggested that mental imagery vividness may be positively associated with sensory sensitivities, possibly through the shared mechanism of hyperexcitability of the sensory cortices. The aim of this paper was to explore this contradictory set of associations across two modalities (visual and tactile). We used standardised questionnaires to measure autistic traits, sensory sensitivities, and mental imagery vividness in a sample evenly comprised of autistic and non-autistic adults (*n* = 595). Higher autistic traits were significantly associated with lower mental imagery (*r* =  − 0.20 and *r* =  − 0.17 for visual and tactile imagery respectively), and a higher incidence of aphantasia was observed in the autistic group compared to the non-autistic group. In addition, higher autistic traits were significantly associated with increased sensory sensitivities (*r* = 0.76). Importantly, we found negligible evidence of an association between mental imagery and sensory sensitivity, even when controlling for autistic traits. In the first study to directly explore autistic traits, sensory sensitivities and mental imagery, we conclude that there is no clear evidence to suggest that mental imagery and sensory sensitivity are related, challenging the idea of shared mechanisms of hyperexcitability of sensory cortex.

**Supplementary Information:**

The online version contains supplementary material available at 10.1038/s41598-026-38574-9.

## Introduction

Mental imagery, the process by which we call to mind the phenomenological properties of stimuli we are not currently experiencing, is an ability which varies markedly between individuals^1^. Some people report imagery which is as vivid as real sensory experiences, while others experience no voluntary imagery—a variation termed aphantasia. Aphantasia has been associated with a range of cognitive and behavioural traits, including lower autobiographical and working memory abilities^2^, and lower imaginative abilities^3,4^. Recent research has also linked aphantasia with elevated autistic traits^3,5,6^. These findings fit within a broader profile of imagination differences associated with autism, such as difficulties with generativity^7^ and engaging in pretend play^8^. Aphantasia has recently been associated with sensory sensitivities^9^, which are part of the diagnostic criteria for autism^10^. This crossover is not currently understood. Note that throughout this paper, sensory sensitivities refer to both hyper- and hypo-sensitivities, unless stated otherwise. Mental imagery vividness, meanwhile, refers to how clear, bright, or intense an internally generated image is Syed et al. (2020) ^10^.

Mental imagery has been conceptualised as a form of “perception in reverse”^11^. Neuroimaging evidence indicates that during visual mental imagery, a reversal in activation of brain areas compared to perception can be observed, with information flowing top-down from frontal areas to the primary visual cortex^12^. Activation of the associated sensory cortices during imagery has also been shown for auditory^13^, olfactory^14^, and tactile mental imagery^15^. The neural correlates of imagery vividness are still unclear (Zeman 2024) ^3^; however, visual imagery has been associated with visual cortical excitability^16,17^. An increased excitation-to-inhibition ratio has also been proposed to underlie unusual responses to sensory input, or subjective sensory sensitivities^18^*.* Based on these theories, Dance et al.^9^ proposed a model linking sensory sensitivity with mental imagery vividness, whereby low excitability in the sensory cortices results in both low mental imagery and low sensory sensitivity. Consistent with this model, they report finding lower levels of hyper- and hypo-sensitivities in aphantasic participants, and a positive association between sensory sensitivities and mental imagery in the general population^9^. However, it is important to note that the influence of autistic traits was controlled for, so it is not clear how they may have impacted the findings.

The role of autistic traits in the association between mental imagery and sensory sensitivity is of interest not only because of the well-established link between autism and sensory sensitivities^19,20^, but also because autistic traits have recently been linked to differences in mental imagery. Three recent papers reported complementary findings: that aphantasics have higher levels of self-reported autistic traits than non-aphantasics^3,5^ and that autistic participants scored lower on measures of visual mental imagery and were more likely to attain scores indicative of aphantasia^6^. Furthermore, a negative association was observed between visual mental imagery and autistic traits across a sample comprised of autistic and non-autistic participants^6^. In contrast to these findings, a study assessing mental imagery and autistic traits in a student population found no association between vividness of mental imagery and autistic traits^21^. Further, despite testing multiple modalities of sensory imagery, King et al.^6^ found that only visual imagery was found to be associated with autism diagnosis. It is possible that autism is associated with extremes of mental imagery (Zeman 2024) ^16^ ; that is, increased incidence of both aphantasia and hyperphantasia, which is unusually vivid mental imagery^2^. More vivid mental imagery has been identified as a potential risk factor for mental health conditions that involve intrusive mental imagery, such as post-traumatic stress disorder (PTSD), obsessive–compulsive disorder (OCD), and bipolar disorder^22^. As the prevalence of many of these conditions are elevated in autistic people compared to the general population^23^, it is important to fully understand how autism and mental imagery are related.

The current findings paint an inconsistent picture of the association between mental imagery, sensory sensitivity, and autistic traits. Given that autistic traits are positively aligned with sensory sensitivities and negatively aligned with mental imagery, we might expect to see a negative association between mental imagery and sensory sensitivity. However, the only study on this topic reported a positive association^9^, but interpretation was limited as autistic traits were controlled for in their analysis. A possible explanation for these theoretically inconsistent findings is that autistic traits moderate the association between mental imagery vividness and sensory sensitivity, such that the association between imagery and sensitivity differs depending on the level of autistic traits.

The purpose of this study was to use questionnaire measures to explore the associations between mental imagery vividness, sensory sensitivity, and autistic traits in a large sample evenly comprised of autistic and non-autistic participants. The majority of mental imagery studies tend to focus solely on visual mental imagery; however, mental imagery can be experienced in any sensory modality^9^. For example, we can have an auditory image of a person’s voice or a tactile image of how sand feels in our hands. Tactile imagery has been found to be the second most frequently affected imagery domain in aphantasic participants^9^, and atypical tactile processing is understudied compared to visual and auditory processing (Cascio et al. 2012) ^26^. Therefore, we aimed to test these associations for both visual and tactile mental imagery. As our primary research goal was to understand the associations between these three constructs, our main approach was to explore the data dimensionally across the whole sample. We predicted higher autistic traits would associate with higher levels of sensory sensitivities and also with lower levels of mental imagery. However, we had no specific prediction about the association between sensory sensitivities and mental imagery. We had two secondary research questions. First, as a possible explanation for the inconsistent findings currently present in the literature, we aimed to test whether levels of autistic traits moderate the association between mental imagery and sensory sensitivity. Finally, in order to gain a broader understanding of the potentially complex nature of imagery vividness in autism, we aimed to test whether autism is associated with extremes of visual mental imagery, that is, a higher incidence of aphantasia or hyperphantasia.

## Methods

### Participants

A total of 595 participants (288 male, 302 female, 5 other, mean age = 37.67, SD = 11.70) were recruited via Prolific, an online platform for recruiting research participants (www.prolific.com). All participants were based in the United Kingdom and were compensated for their time. The study, including all methods, was carried out in accordance with appropriate ethical guidelines, with ethical approval granted by the School of Psychology Research Ethics Committee, Cardiff University. Accordingly, informed consent was obtained from all participants.

We used Prolific’s in built screening features to recruit one sample of participants who reported that they were autistic and one group who reported that they were not autistic. In each group, a balanced sample of male and female participants were recruited. Age- matching was achieved by restricting the age criteria for participants in the non-autistic group based on the age of the autistic participants. This was because initial recruitment of a smaller number of participants revealed that the mean age of the autistic group was lower than that of the non-autistic group. Participants were further asked whether they had a diagnosis of autism with three possible responses (“yes”/ “no”/ “no but I believe I am autistic”). We checked whether their responses matched with their answer to the same question when they originally signed up to Prolific. Twenty-four participants signed up to Prolific as autistic but answered not autistic on our questionnaire. These participants were excluded from group-level analyses but retained for whole-sample analyses. Four participants signed up to Prolific as not autistic but answered autistic on our questionnaire. Two of these provided us with the age they were diagnosed and who diagnosed them. These participants were included as part of the autistic group. The remaining two participants responded “N/A” to the questions regarding circumstances of diagnoses. The CATI scores of these participants were inspected and found to be low (78 and 112—see Table 2 for average scores for each group). Therefore, we made the decision to keep these two participants in the not autistic group.

Demographic information for each of the three groups can be seen in Table 1.

**Table 1:** Demographic information for each group.

	Total (n)	Female (n)	Male (n)	Other (n)	Mean age, years (SD)
Autistic—diagnosed	254	128	124	2	36.9 (11.0)
Autistic—self-identified	61	31	30	0	37.9 (11.3)
Not autistic	256	133	121	2	38.8 (12.5)

### Materials

Our materials consisted of four questionnaires, described below. Within each questionnaire was an attention check question, which asked participants to select a specific answer. These were used to identify participants who may not have been paying attention and so remove them from the analyses.

#### Vividness of visual imagery questionnaire

We used a modified version of Vividness of Visual Imagery Questionnaire (VVIQ) (Marks 1973)^27^ to examine participants’ visual mental imagery. The VVIQ is a 16-item questionnaire which asks participants to consider four different scenarios, and rate how vividly they can imagine four aspects of each scenario. For example, one scenario asks participants to imagine a country scene involving trees, mountains and a lake. They are then asked to rate how vividly they can imagine the following four aspects of the scene: the contours of the landscape, the colour and shape of the trees, the colour and shape of the lake, and a strong wind blowing on the tree and the lake causing waves. Vividness of mental imagery was rated on a 5-point rating scale, where 1 represented “no image at all, you only “know” that you are thinking of the object” and 5 represented imagery which is “perfectly clear and as vivid as normal vision”.

The instructions that we used were based on a variant version of the VVIQ used by Zeman et al.^2^ and further modified for our purposes. Although use of variant versions has been criticised^24^, we made this decision following personal communication with Professor Adam Zeman and feedback from our autistic consultant. In particular, feedback suggested that the original instructions may not be suitable for autistic participants due to length and style. The shorter instructions used by Zeman et al. were considered more accessible and therefore more appropriate for use in our chosen population. We added some minor changes to the instructions for additional clarity.

The total score, which was the summed score across the 16 scenarios, was used in the current study, with scores ranging from 16 to 80.

#### Adapted shortened Betts’ questionnaire upon mental imagery (tactile subscale)

We used the tactile subscale of the Adapted shortened Betts’ questionnaire upon mental imagery (Betts) (Sheehan 1967; Spiller et al. 2015) ^29,30^ to examine strength of tactile imagery. This subscale asks participants to imagine touching 12 different substances or items with their hand. Example items include sand, glass, the warmth of a tepid bath and the prick of a pin. Participants are asked to rate the strength of their tactile imagery on a 5-point scale where 1 corresponds to “no touch at all, you only “know” you are thinking of the tactile sensation” and 5 corresponds to “perfectly realistic and as vivid as the actual tactile sensation”. The total score for this subscale was used, with possible scores ranging from 12 to 60.

#### Glasgow sensory questionnaire

The Glasgow Sensory Questionnaire version 1.2 (GSQ) (Robertson and Simmons 2013) ^31^ was used to measure sensory sensitivity. The GSQ contains 42 items which can be split into seven subscales, each investigating sensory sensitivity in a different sensory modality: visual, auditory, tactile, olfactory, gustatory, vestibular and proprioceptive. Each subscale contains six items, three of which measure hypersensitivity and three of which measure hyposensitivity. Example items include “Do you dislike loud noises?”, “Do you dislike the physical sensation you get when people hug you?” and “Do bright lights ever hurt your eyes/cause a headache?”. A rating scale of 0–4 is used, where 0 is “never” and 4 is “always”. For our primary analysis, we used the total score summed across all items, with possible scores ranging from 0 to 168.

#### The comprehensive autistic trait inventory

The Comprehensive Autistic Trait Inventory (CATI) (English et al. 2021) ^32^ was used to measure autistic traits. The CATI is a 42-item questionnaire comprised of 6 equally-weighted subscales (Social Interactions, Communication, Social Camouflage, Cognitive Rigidity, Repetitive Behaviours and Sensory Sensitivity). Example items include “I like to stick to certain routines for everyday tasks”, “I look for strategies and ways to appear more sociable”, and “Social occasions are often challenging for me.” Responses are scored on a scale of 1 (“Definitely disagree”) to 5 (“Definitely agree”). Five of the 42 items are reverse-scored. We used the total score, summed across all 6 subscales, for comparisons with the mental imagery questionnaires. For comparisons with the GSQ, we removed the sensory sensitivity subscale to avoid artificially high correlations (referred to hereafter as CATI-edited). Possible scores range from 42 to 210 across the whole questionnaire, and 35–175 with the sensory subscale removed.

### Procedure

Participants were directed from Prolific to a survey hosted in Qualtrics, where they were first asked to complete a short demographic questionnaire. They were then asked to complete the two imagery questionnaires (VVIQ and Betts) in a random order. Next, participants completed the remaining five questionnaires in a random order (the GSQ, CATI, and three additional questionnaires measuring anxiety and visual hypersensitivity for use in a different study). The imagery questionnaires were administered first due to the task differences between these and the other questionnaires—the imagery questionnaires required participants to conjure specific mental images, while remaining measures involved reflecting on previous experiences. Participants were provided with a debrief at the end of the study.

### Data analysis

Data preparation and statistical analyses were carried out in RStudio (Posit team, 2023).

#### Data preparation

Eighteen participants were removed: fourteen due to incomplete data and four for failing more than one attention check.

#### Statistical analyses

Histograms were inspected for normality and tests of skew and kurtosis were performed for all measures. There was some evidence of non-normal distributions, so Pearson’s correlations were tested for replication with Spearman’s correlations. All correlations replicated, so only Pearson’s correlations are reported. Correlations were used to establish patterns of association between the key variables: mental imagery (VVIQ, Betts), sensory sensitivity (GSQ), and autistic traits (CATI-edited).

Next, we replicated the analysis method of Dance et al.^9^ by constructing a series of linear regression models, so that our results could be more directly compared. Model 1 predicted sensory sensitivity (GSQ) from autistic traits (CATI-edited). Next, we added visual mental imagery (VVIQ) as an additional predictor (model 2). Finally, for model 3 we added tactile mental imagery (Betts) to model 1.

In order to examine whether autistic traits had a moderating effect on the association between mental imagery and sensory sensitivities, we constructed two further regression models. Model 4 used CATI-edited scores, VVIQ scores, and the interaction effect between these two predictors to predict GSQ scores. Model 5 used CATI-edited scores, Betts scores, and the interaction effect between these two predictors to predict GSQ scores. In both models, predictor variables were mean-centred.

A chi square test of independence examined the association between autism diagnosis and visual mental imagery category. Visual mental imagery categories consisted of aphantasia, midrange imagery vividness, and hyperphantasia. Participants were classified as aphantasic if they scored between 16 and 32 on the VVIQ (Keogh and Pearson 2018) ^33^. Hyperphantasia was considered to be imagery in the realm of 75–80, indicating imagery which is almost as vivid as normal perception^2^. Scores between 33 and 74 were considered to indicate midrange imagery vividness. Only the diagnosed autistic and not autistic groups were included in this association test, due to the smaller sample size of the self-identified autistic group violating the assumption of expected frequencies being above five.

### Participatory methods

An autistic consultant gave feedback on all study materials, advising on appropriateness and clarity.

## Results

### Descriptive statistics

Descriptive statistics are shown in Table 2, showing that all questionnaires produced a range of responses with a good degree of variance. As expected, the diagnosed autistic group have higher mean scores on measures of autistic traits and sensory sensitivity than the non-autistic group.

**Table 2:** Descriptive statistics showing mean, SD (in brackets) and range of response for each questionnaire.

	Visual imagery (VVIQ)	Tactile imagery (Betts)	Sensory sensitivity (GSQ)	Autistic traits (CATI)
Whole sample	50.4 (14.0)	37.7 (11.9)	59.1 (26.8)	134.0 (36.3)
	16–80	12–60	3–150	49–205
Autistic—diagnosed	47.6 (15.4)	35.9 (13.1)	76.5 (22.7)	161.4 (24.2)
	16–80	12–60	9–150	58–205
Autistic—self-identified	50.3 (13.5)	35.7 (12.1)	59.0 (22.3)	138.3 (22.2)
	16–75	12–60	14–117	78–181
Not autistic	52.8 (12.5)	39.6 (10.7)	41.5 (19.5)	106.1 (27.9)
	16–80	12–60	3–150	49–183

VVIQ, Vividness of Visual Imagery Questionnaire; Betts, Adapted shortened Betts’ questionnaire upon mental imagery (tactile subscale); GSQ, Glasgow Sensory Questionnaire; CATI, Comprehensive Autistic Trait Inventory.

### Associations between mental imagery, sensory sensitivity, and autistic traits across the whole sample

**Fig. 1:**
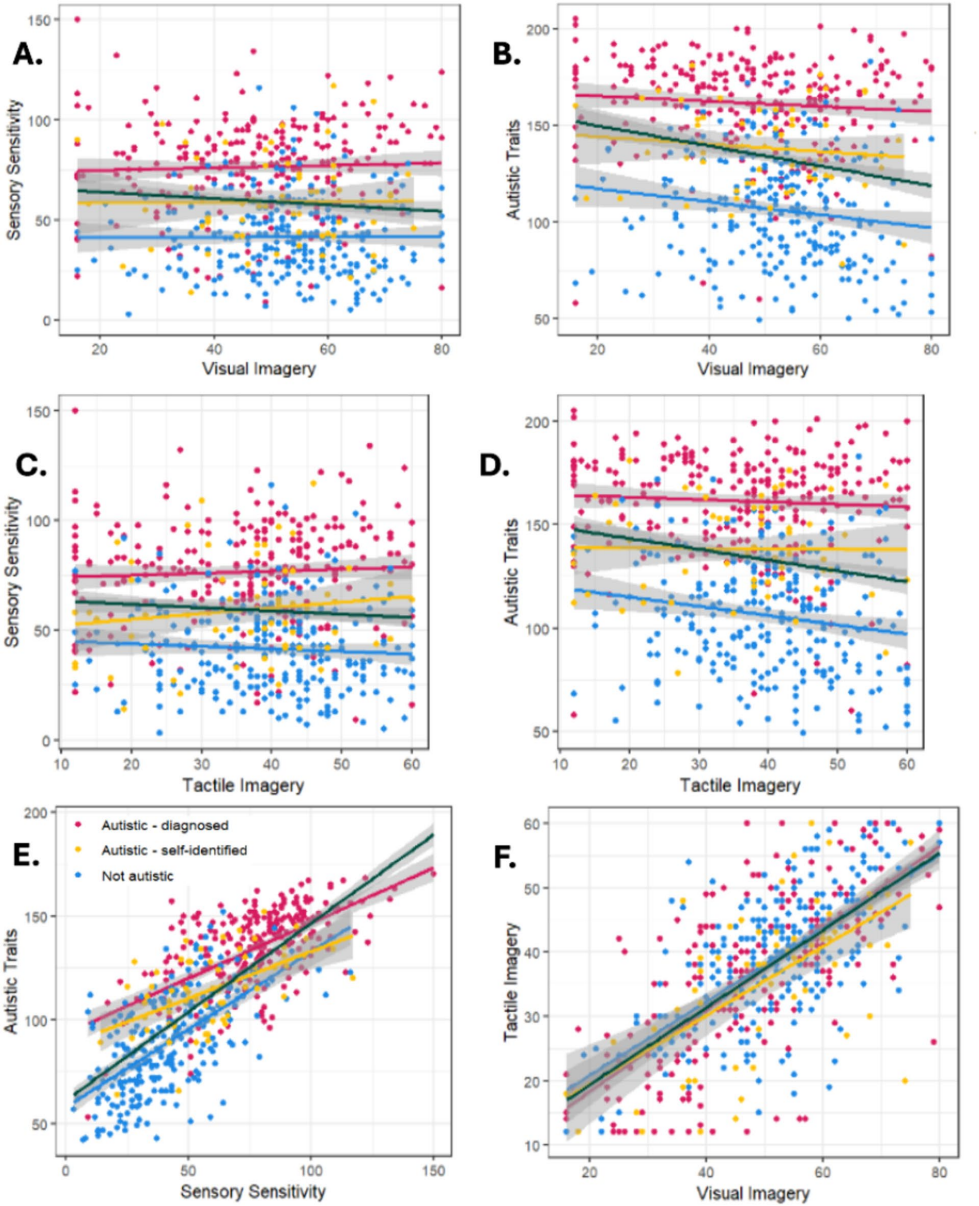
Correlations between visual mental imagery, tactile mental imagery, sensory sensitivity, and autistic traits. This figure shows the correlation between each of the main constructs, with the green linerepresenting the correlation for the whole sample. (**A**) correlation between visual mental imagery (VVIQ) and sensory sensitivity (GSQ). (**B**) correlation between visual mental imagery and autistic traits (CATI). (**C**) correlation between tactile mental imagery and sensory sensitivity. (**D**) correlation between tactile mental imagery (Betts) and autistic traits. (**E**) Correlation between sensory sensitivity and autistic traits (CATI-edited). (**F**) Correlation between visual mental imagery (VVIQ) and tactile mental imagery (Betts). Lines of best fit are shown for each group in their corresponding colour.

The associations between mental imagery, sensory sensitivity, and autistic traits are illustrated in Fig. 1. We observed a significant but very small negative correlation between visual mental imagery and sensory sensitivity (*r* =  − 0.08, *p* = 0.04). If a Bonferroni correction was applied to account for the 5 correlations carried out, this would no longer reach statistical significance (*p* = 0.01). No significant association was observed between tactile mental imagery and sensory sensitivity (*r* =  − 0.07, *p* = 0.1). We also found a significant negative association with moderate effect size between autistic traits and both visual mental imagery (*r* =  − 0.20, *p* < 0.001) and tactile mental imagery (*r* =  − 0.17, *p* < 0.001). As expected, a significant correlation with a large effect size was seen between sensory sensitivity and autistic traits (*r* = 0.76, *p* < 0.001).

As a post-hoc analysis, we explored the modality-specific correlations between imagery and sensitivity (i.e. the correlations between visual imagery and the visual subscale of the GSQ, and tactile imagery and the tactile subscale of the GSQ). No meaningful within-modality correlation was found. Finally, for completeness, we compared visual and tactile mental imagery. A strong correlation was seen here (*r* = 0.71, *p* < 0.001).

In order to directly compare our data to that of Dance et al.^9^, we replicated their analysis approach, using regression to compare a model predicting sensory sensitivity from autistic traits alone (Model 1), to models predicting sensory sensitivity from autistic traits and visual mental imagery (Model 2) or from autistic traits and tactile mental imagery (Model 3; see Methods for details).

Model 2 accounted for only 0.5% more of the variance than model 1 (58.7% compared to 58.2%, Δ*R*^2^ = 0.005)). Model 3 accounted for 0.4% more of the variance than model 1 (58.6% compared to 58.2% (Δ*R*^2^ = 0.004)). In models 2 and 3, visual and tactile mental imagery were significant, but only slight, positive predictors of sensory sensitivity. Coefficients are provided in Table 3.

**Table 3:** Coefficients for regression models 1–3, illustrating the contribution of mental imagery to sensory sensitivity (GSQ) when autistic traits are controlled.

Predictor	*b*	SE(*b*)	*t*	*P*
Model 1				
(Intercept)	− 17.31	2.75	− 6.30	< 0.001
Autistic traits (CATI-edited)	0.68	0.02	28.79	< 0.001
Model 2				
(Intercept)	− 25.89	4.16	− 6.23	< 0.001
Autistic traits (CATI-edited)	0.70	0.02	28.90	< 0.001
Visual mental imagery (VVIQ)	0.14	0.05	2.74	0.006
Model 3				
(Intercept)	− 24.10	3.87	− 6.24	< 0.001
Autistic traits (CATI-edited)	0.70	0.02	28.91	< 0.001
Tactile mental imagery (Betts)	0.15	0.06	2.49	0.013

VVIQ, Vividness of Visual Imagery Questionnaire; Betts, Adapted shortened Betts’ questionnaire upon mental imagery (tactile subscale); GSQ, Glasgow Sensory Questionnaire; CATI, Comprehensive Autistic Trait Inventory.

Therefore, although we found that correlation between visual mental imagery and sensory sensitivity is negative, and no correlation is observed between tactile mental imagery and sensory sensitivity (see Fig. 1), when autistic traits are accounted for there is a significant positive association between both types of mental imagery and sensory sensitivity. However, as with the initial correlations, these effects are very weak, and the percentage of additional variance models 2 and 3 account for when compared to model 1 is very low.

### Do autistic traits moderate the association between mental imagery and sensory sensitivity?

To test whether autistic traits moderate the association between mental imagery and sensory sensitivity, we constructed further regression models predicting sensory sensitivity from autistic traits, mental imagery, and the interaction effect between autistic traits and mental imagery (Models 4 and 5; see Methods for details.)

**Fig. 2:**
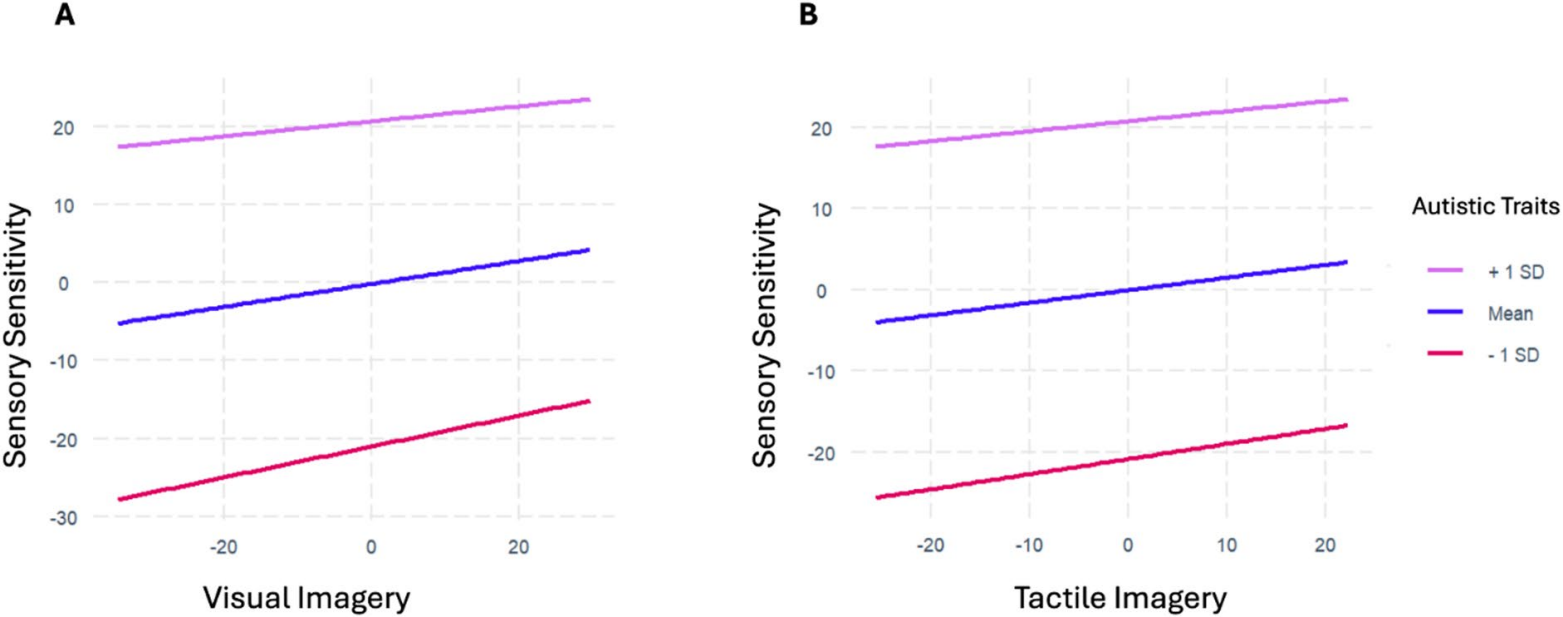
The association between mental imagery and sensory sensitivity at different levels of autistic traits. This figure illustrates the lack of interaction between mental imagery. (**A**) Visual imagery (VVIQ) and (**B**) Tactile imagery (Betts) and autistic traits (CATI-edited). The association between mental imagery and sensory sensitivity at three levels of autistic traits (the mean whole group score, 1 standard deviation above the mean, and one standard deviation below the mean) are shown. These lines are close to parallel, which reflects the non-significant interactions and indicates that the strength, form, and direction of this association do not change based on levels of autistic traits.

We found no evidence of an interaction effect between autistic traits (CATI-edited) and visual mental imagery (VVIQ) (b =  − 0.002, *p* = 0.26). We also found no evidence of an interaction effect between autistic traits and tactile mental imagery (Betts) (b =  − 0.001, *p* = 0.57) (see Fig. 2).

### Comparing incidence of imagery status (aphantasic, midrange, hyperphantasic) in the autistic and non-autistic groups

A chi square test of independence found a significant association between autism diagnosis and imagery status, χ^2^ (2) = 18.83, *p* < 0.001, v = 0.192 (see Table 4). More autistic people were in the aphantasic category and fewer in the midrange imagery vividness category. There was negligible difference in the number of hyperphantasics in the two groups.

**Table 4:** Number of autistic and non-autistic participants in each imagery category.

	Aphantasic	Midrange imagery	Hyperphantasic
Autistic	46	199	9
Non-autistic	15	234	7

### Exploratory analyses of the differential pattern of VVIQ scores across different visual imagery scenarios

As an exploratory post-hoc analysis, we investigated whether there were group differences in the pattern of responses to each of the four scenarios that comprise the VVIQ (see supplementary materials).

## Discussion

Using an online population of autistic and non-autistic adults, this is the first study to directly characterise the associations between mental imagery, sensory sensitivity, and autistic traits in the same sample. A key motivation was to explore the inconsistencies present in the literature. Critically, our findings demonstrated a lack of meaningful association between mental imagery vividness and self-reported sensory sensitivities. This contradicts previous empirical research and the theory that hyperexcitability of the sensory cortex is a shared neural underpinning of these two phenomena^9^. In addition and as expected, we found that lower mental imagery vividness and greater sensory sensitivities both correlated with higher levels of self-reported autistic traits. Our results present a coherent set of associations between the three constructs: while the association between mental imagery and sensory sensitivity was very weak, its direction was consistent with theoretical prediction of a negative relationship.

The main association of interest in this study was the correlation between mental imagery and sensory sensitivity. Given the well-established positive association between autistic traits and sensory sensitivity (e.g. Robertson and Simmons 2013 ^31^; Tomchek and Dunn^19^), and growing evidence indicating a negative association between autistic traits and mental imagery^3,5,6^, we would predict a negative association between sensory sensitivity and mental imagery. However, the only paper to have studied this reported a positive association^9^. In contrast to this previous work, while we did find a significant negative association between visual mental imagery and sensory sensitivities, the effect size was negligible (*r* =  − 0.084), and the correlation between sensory sensitivity and tactile imagery was not significant. Note that these absent or very small correlations cannot be explained by data quality (excessive noise in the online questionnaire measures), because both sensory sensitivity and imagery measures had very strong correlations with other measures: sensory sensitivity with autism traits, and visual imagery with tactile imagery, as expected from previous literature (e.g. Robertson and Simmons 2013; Bilzer and Monzel 2025; Lima et al. 2015) ^31,34,35–38^.

The positive association reported by Dance et al.^9^, focused only on visual mental imagery scores and was found in both in the general population and in an aphantasic sample. Their findings were interpreted as lending support to a cortical excitability model, whereby hyperexcitability in the sensory cortices resulted in heightened sensory sensitivity and more vivid mental imagery^9^. However, in this study the influence of autistic traits was controlled for. When we followed this analytic method, we also found that both visual and tactile imagery scores were significant positive predictors of sensory sensitivity. However, this effect was again negligible, with imagery scores accounting for between 0.4 and 0.5% of the variance in sensory sensitivity scores (Δ*R*^2^ = 0.005 and 0.004 for visual and tactile imagery, respectively). In Dance et al.’s study, the effect was also weak, with visual imagery scores predicting 3.9% of the variance. Taken together, these two studies present little evidence of a meaningful association between sensory sensitivity and mental imagery and as such, little evidence of cortical excitability constituting a shared mechanism underlying these two constructs. Instead, our finding implies dissociable mechanisms between mental imagery vividness and sensory sensitivity. This may be because generation of mental images is an entirely top-down process that does not rely on concurrent sensory input. Sensory sensitivity, meanwhile, occurs in the presence of sensory stimulation. While it is unclear to what extent sensory sensitivity may rely on top-down mechanisms such as attention^18^, unlike mental imagery, it occurs in the context of bottom-up stimulus processing.

Existing research into the association between mental imagery and autism has indicated that higher autistic traits may be associated with lower visual mental imagery^3,5,6^. Our findings support this and extend the findings to tactile mental imagery, albeit with a small effect size. Across our whole sample, we found negative correlations between both mental imagery domains and autistic traits (*r* =  − 0.2 and *r* =  − 0.17 for visual and tactile imagery, respectively). This finding is broadly comparable with the one previous study which explored the association between visual mental imagery and autistic traits dimensionally (*r* =  − 0.31; King et al.^6^). We also found that autistic participants were more likely to attain scores indicative of aphantasia than non-autistic participants.

Our results differ from previous findings in that we report a significant association between tactile mental imagery and autistic traits. Only one study has investigated whether group differences in tactile imagery vividness exist between autistic and non-autistic participants^6^, reporting no significant difference. However, to our knowledge, this association has not been tested dimensionally. As our finding was relatively weak (*r* =  − 0.17), it is possible that this would not manifest as a difference at the group level.

Our findings indicate that while lower mental imagery is related to elevated autistic traits, it is not through a shared association with elevated sensory sensitivities. This raises the question: which aspect of autism is mental imagery associated with? An obvious candidate would be via general difficulties with imagination. Although findings are mixed^5^, differences in or difficulties with various aspects of generativity and imaginative ability are commonly associated with autism and were included in part of the original “triad of impairments” thought to define autism^25^. While mental imagery and imagination are different concepts, they are related^5^, and therefore it is likely that difficulties with imagination and lower mental imagery vividness are associated in autism. Another possibility is that reported mental imagery vividness is related to differences in communication. Specifically, differences in pragmatic reasoning between autistic and non-autistic people^26^ may result in a more literal interpretation of imagery tasks in the autistic group. For example, they may be less likely to interpret that their imagery is as vivid as normal perception, as it is not “real seeing” or “real touch”. Both of these interpretations would fit with the finding that of the five subscales of the Autism Spectrum Quotient^27^, aphantasic participants only scored lower on the imagination and social skills subscale compared to non-aphantasic participants^5^.

The growing evidence of a negative association between voluntary mental imagery and autistic traits may have important clinical applications. Several therapeutic practices used in the treatment of mental illness, particularly anxiety disorders, use mental imagery as a key part of their approach^22^. For example, a patient with OCD who carries out compulsive cleaning rituals may be treated using imaginal exposure, whereby they imagine themselves having contaminated hands, but not washing them, and maintain this mental scenario until their anxiety decreases^22^. A patient with social anxiety may be treated using imagery rescripting, where they are required to imagine a stressful scenario, such as performing poorly in public speaking, and change the content of the scenario such that they are performing well^22^. As many mental illnesses are associated with negative and intrusive mental images, these imagery-based techniques are an important aid to traditional talking-based therapies, which may only target the verbal content of disordered thinking. More generally, mindfulness-based meditation techniques are commonly recommended by general practitioners and mental health professionals to target symptoms of stress or anxiety. Mindfulness meditation involves paying attention to the present and observing thoughts without judgment, and often involves guided imagery practices that can engage a range of senses (Kharlas and Frewen 2016) ^39,40–43^. However, techniques that rely on mental imagery may be less effective in participants with low or no mental imagery^28^. As rates of many mental illnesses are known to be elevated in autism^23^, understanding the relation between autistic traits and mental imagery may be an important consideration in future research into the efficacy of these therapies in different groups. More generally, a greater awareness of the variability in mental imagery vividness might allow for certain approaches, such as mindfulness meditations, to be adapted and tailored to participants’ individual abilities and needs. However, it must be emphasised that we observed the full spectrum of mental imagery vividness in all of our groups. Furthermore, our study relied on self-reported measures (the limitations of which are discussed in the “Limitations” section), and the association between mental imagery and autistic traits was moderate (*r* =  − 0.20 and *r* =  − 0.17 for visual and tactile imagery, respectively). Therefore, while the influence of autistic traits should be considered in future research, in therapeutic scenarios the appropriateness of such treatments should be considered on a case-by-case basis.

As very little is known about visual imagery in autism, it is possible that autistic people may find imagining particular types of scenario difficult. As such, we conducted exploratory post-hoc analysis (see supplementary materials) to see if there were differences in responses to each of the four VVIQ scenarios depending on autism diagnosis. Two of the scenarios required participants to draw on their memory of either a person or a shop to create a visual image, whereas the other two scenarios were of an imagined natural scene, which could be fictitious. Given noted imagination and generativity differences in autism^7,29^, it is possible that there may be performance differences based on the type of imagery required. Furthermore, prosopagnosia is associated with higher autistic traits^30^, which suggested that there may be group differences in performance on the scenario requiring participants to visualise a person they know. However, we found that while there was an effect of both question and group, there was no interaction between the two. This indicates that while the autistic group scored lower on average than the non-autistic group, the pattern of performance was similar between groups. This finding also underlines that systematic differences in the ability to imagine different types of visual scenarios are unlikely to have affected our pattern of findings.

### Limitations

One difficulty with researching mental imagery differences is that we are measuring experiences that are entirely private. Judgements of these experiences are open to error and may be influenced by a variety of factors^2^. It is possible that there is an association between mental imagery vividness and sensory sensitivity, but we did not find it due to people comparing their imagery to their own sensory experiences. For example, two people with differing levels of sensory sensitivities may have both rated their imagery as “clear and reasonably vivid”, because their mental image is a clear imitation of their sensory experience. However, as their sensory experiences are different, their mental imagery vividness may also be very different, and each is unable to imagine the other’s sensory or mental experiences. However, previous research has found that the fMRI BOLD signal observed across a widespread network of brain regions when people are engaging in mental imagery tasks correlates with self-reported visual imagery vividness using the VVIQ^31^, which suggests that people do have good insight into their own mental imagery.

Relatedly, but more broadly, this work relies entirely on self-report measures. This is necessary due to the subjective and internal nature of the constructs that we are examining. Currently, objective measures do not exist for mental imagery vividness: measures such as the Mental Rotation Task (MRT; Shepard and Metzler 1971) ^44^ can be considered more objective measures of mental imagery abilities, but they do not probe vividness specifically, and may rely on other processes such as working memory abilities (Pounder et al. 2022) ^45^ . Similarly, self-reported sensory sensitivity generally has a poor correlation with more objective measures such as detection or discrimination tasks (Schulz and Stevenson 2021) ^46,47^, suggesting that the subjective feeling of over- or under-responsiveness to sensory input is distinct from psychophysical performance. Nevertheless, as this study relies solely on questionnaire data, it is possible that the weak associations found between, for example, visual mental imagery and sensory sensitivity, can be accounted for by differences in individual participants’ interpretation of the questions.

### Future directions

A recommendation for future work is to better understand the way that mental imagery and mental illnesses are associated in autistic people. Despite a negative association between autistic traits and vividness of mental imagery, autistic people are more at risk of developing conditions associated with intrusive imagery^23^. Future research could investigate if low imagery is a protective factor against these conditions in autistic people, or if they way that autistic people with low imagery vividness experience these conditions differ. For example, an autistic person with low imagery vividness may still have an elevated risk of developing OCD, but this is more likely to manifest as obsessive thoughts surrounding an idea, rather than the intrusive images reportedly experienced by 81% of those with OCD^32^. These questions are important, both for understanding risk factors and developing treatment strategies.

The measures used in this study, consistent with the majority of studies investigating mental imagery^24^, required participants to conjure mental images of benign, everyday scenarios or objects. It is conceivable that while this type of imagery is not associated with sensory sensitivities, imagining sensory stimuli that commonly trigger sensitivities—for example, uncomfortable visual patterns or clothing textures—could. This would make sense in light of previously discussed findings that negative imagery is prevalent in a range of psychopathologies^22^. Future research could consider measuring mental imagery abilities of clinically- or situationally- relevant scenarios, objects or sensations.

## Conclusion

We carried out this study to explore a conflicting set of reported associations between mental imagery, sensory sensitivity, and autistic traits. We have demonstrated that this conflicting set of findings can be explained by a lack of meaningful association between mental imagery and sensory sensitivity. We found evidence of an association between autistic traits and mental imagery, which could have implications for therapeutic treatments for mental illnesses which utilise imagery-based techniques.

## Supplementary Information

Below is the link to the electronic supplementary material.

Supplementary Material 1

## Data Availability

The data are available from the corresponding author on reasonable request.
