# Supplementary material for: Exploring the association between mental imagery, sensory sensitivity, and autistic traits in autistic and non-autistic adults
Source: Sci Rep. 2026 Feb 25;16:11018. doi: 10.1038/s41598-026-38574-9 (PMC13044261; doi:10.1038/s41598-026-38574-9)
Supplement: Supplementary file 1 — Supplementary Material 1 [file 41598_2026_38574_MOESM1_ESM.docx]

**Correlations between cross-modality mental imagery abilities, and within-modality mental imagery and sensory sensitivity**

Supplementary Table 1: Correlations between visual mental imagery, tactile mental imagery, visual sensory sensitivity and tactile sensory sensitivity.

|  | 1 | 2 | 3 |
| --- | --- | --- | --- |
| 1. Visual mental imagery (VVIQ) |  |  |  |
| 2. Tactile mental imagery (Betts) | 0.71*** |  |  |
| 3. Visual sensory sensitivity (GSQ: visual subscale) | -0.05 | -0.01 |  |
| 4. Tactile sensory sensitivity (GSQ: tactile subscale) | -0.09* | -0.08* | 0.72*** |
| ****p<0.001, **p<0.01, *p<0.05* | | | |

*VVIQ = Vividness of Visual Imagery Questionnaire, Betts = Adapted shortened Betts’ questionnaire upon mental imagery (tactile subscale), GSQ = Glasgow Sensory Questionnaire*

**Exploratory Analyses of the differential pattern of VVIQ scores across different visual imagery scenarios**

The four scenarios in the VVIQ that make up the visual imagery score probe different types of imagination. For example, some scenarios rely more heavily on memory while others may rely more on generative ability; some explicitly require visual imagery of people whilst others are not inherently social. An exploratory post-hoc 3 (autism diagnosis group) x 4 (VVIQ scenario) analysis of variance (ANOVA) probed whether there were group differences in the pattern of responses to individual scenarios. This revealed a main effect of group (F[2,2272]=26.47, p<0.01) and VVIQ scenario (F[3,2272]=5.83, p<0.01), but no interaction between group and scenario (F[6,2272]=0.68, p>0.05). This suggests consistency in the relative pattern of performance across the scenarios for the different diagnostic groups. Descriptive statistics can be seen in Supplementary Table 2.

Supplementary Table 2: Descriptive Statistics showing mean and SD (in brackets) scores for each VVIQ scenario.

| VVIQ Scenario | | | | |
| --- | --- | --- | --- | --- |
|  | 1 | 2 | 3 | 4 |
| Autistic - diagnosed | 11.4(4.3) | 12.5(4.3) | 12.2(4.1) | 11.6(4.4) |
| Autistic - self-identified | 12.7(3.9) | 13.3(3.9) | 12.1(4.0) | 12.2(4.1) |
| Not autistic | 13.1(3.8) | 13.6(3.6) | 13.3(3.6) | 12.8(3.8) |

**Exploratory Analyses of the differential pattern of VVIQ scores across different visual imagery scenarios: Pairwise follow up comparisons**

Pairwise follow up comparisons with Bonferroni correction revealed significant differences between scenario 1 (person) and 2 (natural scene: rising sun) (t(2280) = -3.39, adjusted p=0.004), and scenario 2(natural scene: rising sun) and 4 (natural scene: lake) (t(2280) = 3.61, adjusted p=0.002). This indicates that on average, participants scored higher on scenario 2 (natural scene: rising sun) than scenario 1 (person) and scenario 4 (natural scene: lake).
